# Algorithm for the Time-Propagation of the Radial Diffusion Equation Based on a Gaussian Quadrature

**DOI:** 10.1371/journal.pone.0132273

**Published:** 2015-07-24

**Authors:** Dirk Gillespie

**Affiliations:** Department of Molecular Biophysics and Physiology, Rush University Medical Center, 1750 West Harrison Street, Suite 1289, Chicago, Illinois, United States of America; University of Catania, ITALY

## Abstract

The numerical integration of the time-dependent spherically-symmetric radial diffusion equation from a point source is considered. The flux through the source can vary in time, possibly stochastically based on the concentration produced by the source itself. Fick’s one-dimensional diffusion equation is integrated over a time interval by considering a source term and a propagation term. The source term adds new particles during the time interval, while the propagation term diffuses the concentration profile of the previous time step. The integral in the propagation term is evaluated numerically using a combination of a new diffusion-specific Gaussian quadrature and interpolation on a diffusion-specific grid. This attempts to balance accuracy with the least number of points for both integration and interpolation. The theory can also be extended to include a simple reaction-diffusion equation in the limit of high buffer concentrations. The method is unconditionally stable. In fact, not only does it converge for any time step Δ*t*, the method offers one advantage over other methods because Δ*t* can be arbitrarily large; it is solely defined by the timescale on which the flux source turns on and off.

## Introduction

Diffusion is one of the most classic problems in physics, with the governing laws of Fick dating back more than 150 years [1, 2] and a number of books on the subject [3, 4, 5]. From both the mathematical and numerical points of view, the diffusion equation—especially the spherically-symmetric version considered here—is very well studied; its mathematical properties are known and a number of numerical techniques for solving it exist (e.g., explicit-time methods like forward-time/central-space (FTCS), implicit-time methods like backward-time/central-space (BTCS) and Crank-Nicolson, and more sophisticated ones [6]). While diffusion has been studied experimentally and theoretically for a very long time, it is still of central importance in many areas today. For example, it plays vital roles in biology [7] and new technologies like nanofluidics [8].

For these applications, the efficient numerical solution of the diffusion equation is fundamental to understanding the physics underlying these new applications. One important example is calcium-induced calcium release (CICR) in cardiac muscle, where an array of Ca^2+^-selective ion channels open and close with a probability that depends on the local Ca^2+^ concentration, which comes from the diffusion of Ca^2+^ through neighboring channels [9]. In this sense, the channel’s open/closed state history affects its future state in a feedback mechanism. For such systems, the source flux is changing stochastically in time, depending on the on/off states of all the other flux sources. Moreover, since each channel is ∼ 40 nm across, the calculation of a 10 × 10 array of these channels must be accurate over long distances (at least 560 nm) to understand the complex interactions.

Traditional numerical algorithms like BTCS and Crank-Nicolson divide the space coordinate into small slices to calculate numerical derivatives and produce a matrix equation that must be solved at each time step. Similarly, the conditionally-stable explicit-time methods like FTCS require a matrix/vector multiplication. Because the matrix is sparse, the number of flops for this solve is proportional to the number of grid points. These approaches are very valuable and easy to implement, but when the number of grid points reaches well into the thousands for large systems other approaches might be more computationally efficient.

Here, one alternative numerical technique to compute the concentration profile for radial Fickian diffusion from a point source is described. Specifically, the spherically-symmetric equation considered here is

$$\begin{array}{c} \frac{\partial c(r,t)}{\partial t}=\frac{D}{r^{2}}\frac{\partial }{\partial r}(r^{2}\frac{\partial c}{\partial r}(r,t))+s(r,t) \end{array}$$ (1)

with the initial condition

$$\begin{array}{c} c(r,0)=c_{0}, \end{array}$$ (2)

which we take to be a constant. Here, *c*(*r*, *t*) is the concentration (number per unit volume) of the diffusing particles and *s*(*r*, *t*) is the flux per volume (number per unit volume per time) injected into the system. Here, a point source is considered. *D* is the diffusion coefficient and the variables *r* and *t* are the distance from the origin and time, respectively.

The purpose of this paper is describe a method to solve Eq (1) numerically for *c*(*r*, *t*). Instead of discretizing the differential equation itself, the propagation algorithm described here is based on an integral version of the solution to Eq (1). This approach splits the solution into two components, the diffusion from the source during the time interval (whose exact solution is known) and the propagation of the particles released during previous time intervals. Here, we show that the propagation integral can be very efficiently calculated using a combination of (1) a new Gaussian quadrature that is specifically formulated for the diffusion equation and (2) interpolation with grid points whose locations are chosen based on the exact solution to the diffusion equation. This, then, minimizes both the number of points needed to evaluate the integral and the number of points needed to interpolate the results between grid points.

The propagation method offers advantages over more classical techniques. First, since no numerical time derivatives are used, the time step Δ*t* does not have to be small. In the propagation method, Δ*t* is determined by the source’s timescale. For example, if the source flux varies smoothly, then Δ*t* is chosen to be small to approximate that function. However, if the source turns on and off stochastically, then Δ*t* should be the timescale that the source operates on; if, for example, it changes at most once a second, then Δ*t* is 1 second. Second, since no numerical spatial derivatives are used with a constant Δ*r*, very large numbers of grid points (and therefore very large tridiagonal matrices) are avoided. In the propagation method, a nonuniform grid is created that places points optimally for the diffusion problem and between those points interpolation is shown to be accurate.

Preliminary calculations show that a 10-point Gaussian quadrature and ∼ 100 interpolation grid points can give very accurate results, even over long simulations using only a matrix/vector multiplication with a sparse matrix containing < 1400 nonzero entries. These calculations also show that it is possible to evaluate the error of the propagation technique. Moreover, the technique is fast enough to find an optimal set of simulation parameters that maximize accuracy and minimize calculation time. This error and parameter determination is part of the overhead of the simulation so that simulations of the system of interest can be done knowing that the error in the concentration is always within acceptable limits and that the run time is the shortest possible.

## Theory

### Propagation integral formulation

For the source flux in Eq (1) we consider a point source. When the point source has a constant flux *j* that does not vary in time so that

$$\begin{array}{c} s(r,t)=j\delta (r)/4\pi r^{2} \end{array}$$ (3)

where *δ*(*r*) is the Dirac delta function, a simple closed form formula exists in terms of the complementary error function [3]:

$$\begin{array}{c} c(r,t)=\frac{j}{4\pi D}\frac{1}{r}\text{erfc}(\frac{r}{2\sqrt{Dt}}) \end{array}$$ (4)

where

$$\begin{array}{c} \text{erfc}(x)=1-\frac{2}{\sqrt{\pi }}\int_{0}^{x}e^{-t^{2}}dt. \end{array}$$ (5)

However, when the source is not constant over time, Eq (1) must be solved numerically.

One way to attack this problem is via classic finite difference or finite elements methods. Methods like FTCS, BTCS, and Crank-Nicolson produce matrix equations to be solved for the concentration at previously-defined spatial grid points. The choice of grid points is crucial since minimizing the number of points is critical for fast calculation, but accuracy may suffer if the number is too small. The goal of this paper is to provide an alternative solution method that is not based on finite differences or finite elements. This approach propagates the concentration profile in time with a sparse-matrix/vector multiplication, as compared to solving a sparse-matrix equation. Since the grid points in this paper are based on a Gaussian quadrature and an interpolation scheme that are both specifically developed for this diffusion problem, the number of grid points and their locations are optimized in the sense of producing a high-order solution with a minimum number of points.

The approach we use here involves decomposing the concentration into two parts, a source part that adds new particles from the point source during the discretized time interval *T*_*n*_ = (*t*_*n*_, *t*_*n*+1_) and a propagation part that diffuses the concentration profile of the last time step through the time interval *T*_*n*_ [3]. That is,

$$\begin{array}{c} c(r,t_{n+1})=c_{\text{prop}}(r,t_{n+1})+c_{\text{source}}(r,t_{n+1}) \end{array}$$ (6)

where

$$\begin{array}{c} c_{\text{prop}}(r,t_{n+1})=\int_{{\mathbb{R}}^{3}}{\left(4\pi D(t_{n+1}-t_{n})\right)}^{-3/2}\exp(-\frac{{\left|r-r^{\prime }\right|}^{2}}{4D(t_{n+1}-t_{n})})c(r^{\prime },t_{n})dr^{\prime } \end{array}$$ (7)

and

$$\begin{array}{ccc} c_{\text{source}}(r,t_{n+1}) & = & \chi_{n}j_{n}\int_{t_{n}}^{t_{n+1}}\int_{{\mathbb{R}}^{3}}{\left(4\pi D(t_{n+1}-t^{\prime })\right)}^{-3/2}\exp(-\frac{{\left|r-r^{\prime }\right|}^{2}}{4D(t_{n+1}-t^{\prime })})s(r^{\prime },t^{\prime })dr^{\prime }dt^{\prime } \end{array}$$ (8)

$$\begin{array}{ccc} & = & \frac{\chi_{n}j_{n}}{4\pi D}\frac{1}{r}\text{erfc}(\frac{r}{2\sqrt{D\Delta t}}) \end{array}$$ (9)

where *χ*_*n*_ is 0 or 1 depending if the source is off or on during the time interval *T*_*n*_, respectively, *j*_*n*_ is the flux during *T*_*n*_, and Δ*t* = *t*_*n*+1_ − *t*_*n*_ is assumed to be the same for all *n*. These classic formulas may be found, for example, in Section 8.4 of the book by Barton [3]. (Note that for a half-space problem like flux through an ion channel on one side of a membrane, the denominator includes 2*π* not 4*π* because the surface area is half that of the whole sphere.)

Here, the source flux takes the form

$$\begin{array}{c} s(r,t)=\chi (t)j(t)\delta (r)/4\pi r^{2} \end{array}$$ (10)

and is assumed to be constant during the discretized time intervals:

$$\begin{array}{c} j(t)=j_{n}\;\forall t\in [t_{n},t_{n+1}). \end{array}$$ (11)

Similarly, *χ*_*n*_, the on or off state of the source, is the same during the entire time interval [*t*_*n*_, *t*_*n*+1_). Note that here we do not assume that the *χ*_*n*_ are known beforehand; for that, an analytic solution of the diffusion equation exists (see Eq (54) later). Here, the on/off state of the source can vary due to random inputs that depend on the concentration profile produced by the source. For example, in CICR, the concentration profile produced by one channel affects the open state of its neighbors, which in turn produce Ca^2+^ profiles that affect the original channel.

It is also important to note that while we assume that *j* is constant over a time interval, that does not mean we are only considering fluxes that are either on or off or that *j* must be the same during all time intervals. Specifically, if the source flux is a smoothly varying function, the time intervals *T*_*n*_ should be chosen small enough so that *j*(*t*) is well-approximated by the piecewise-constant function *j*(*t*) = *j*_*n*_ ≡ *j*(*t*_*n*_) if *t* ∈ *T*_*n*_. In the notation used here, *j*_*n*_ is the flux during the *n*-th time interval and *χ*_*n*_ is a random variable that takes values 0 or 1. The *χ* function is convenient for the case of a randomly on or off source. For a smoothly varying source on the other hand, *χ*_*n*_ = 1 for all *n* and the *j*_*n*_ are different for different *n*.

Since the source term is the concentration profile due to the *time-independent* source flux during the time interval *T*_*n*_, Eq (9) is just Eq (4) applied to this time interval. In Eqs (7) and (8), the vectors **r** and **r**′ (with lengths *r* and *r*′, respectively) are three-dimensional locations near the point source. The propagation integral can be simplified by using the relations

$$\begin{array}{ccc} & & \int_{{\mathbb{R}}^{3}}\exp(-\frac{{\left|r-r^{\prime }\right|}^{2}}{4D\Delta t})c(r^{\prime },t_{n})dr^{\prime }\\ & & \;=2\pi \int_{0}^{\infty }\int_{0}^{\pi }{\left(r^{\prime }\right)}^{2}\sin(\theta^{\prime })\exp(-\frac{r^{2}+{\left(r^{\prime }\right)}^{2}-2rr^{\prime }\cos(\theta^{\prime })}{4D\Delta t})c(r^{\prime },t_{n})d\theta^{\prime }dr^{\prime } \end{array}$$ (12)

and

$$\begin{array}{c} \int_{0}^{\pi }\sin(\theta^{\prime })\exp(\frac{rr^{\prime }\cos(\theta^{\prime })}{2D\Delta t})d\theta^{\prime }=\frac{4D\Delta t}{rr^{\prime }}\sinh(\frac{rr^{\prime }}{2D\Delta t}) \end{array}$$ (13)

to give

$$\begin{array}{c} c_{\text{prop}}(r,t_{n+1})=\frac{1}{\sqrt{\pi D\Delta t}}\frac{1}{r}\exp(-\frac{r^{2}}{4D\Delta t})\int_{0}^{\infty }c(r^{\prime },t_{n})\cdot r^{\prime }\exp(-\frac{{\left(r^{\prime }\right)}^{2}}{4D\Delta t})\sinh(\frac{rr^{\prime }}{2D\Delta t})dr^{\prime }. \end{array}$$ (14)

The accurate integration of this integral is computationally difficult because it requires knowing the function *c*(*r*′, *t*_*n*_) for all *r*′ from 0 to ∞. This is generally difficult because one needs at least some prior knowledge about a reasonable finite upper limit and about acceptable grid spacings for the *r*′. One purpose of this paper is to develop an order *N* integration scheme for this propagation integral using a Gaussian quadrature.

One important thing to note is that Eqs (9) and (14) are exact solutions of the diffusion equation over a time step Δ*t*. Therefore, Δ*t* can be arbitrarily large. Importantly, it is not constrained by having to be small to numerically approximate a time derivative, as it must be in other methods like FTCS, BTCS, and Crank-Nicolson. Δ*t* is then defined only by the time course of the flux source, the rate at which it turns on and off. This is one way that the propagation method provides an advantage over other existing alternatives. Moreover, because Δ*t* can be anything, the propagation method is unconditionally stable.

### Non-dimensionalization

The first step is non-dimensionalizing *r* with the diffusion lengthscale by defining

$$\begin{array}{c} R=\frac{r}{2\sqrt{D\Delta t}} \end{array}$$ (15)

with a similar definition for *R*′. One can then define

$$\begin{array}{c} \rho (R,t_{n})=R\cdot c(2\sqrt{D\Delta t}R,t_{n}) \end{array}$$ (16)

(and similarly for *ρ*_prop_(*R*, *t*_*n*_) and *ρ*_source_(*R*, *t*_*n*_)) so that the propagator integral (14) becomes

$$\begin{array}{c} \rho_{\text{prop}}(R,t_{n+1})=\int_{0}^{\infty }\rho (R^{\prime },t_{n})W(R,R^{\prime })dR^{\prime } \end{array}$$ (17)

with the weight function

$$\begin{array}{ccc} W(R,R^{\prime }) & = & \frac{2}{\sqrt{\pi }}\exp(-R^{2})\exp(-{\left(R^{\prime }\right)}^{2})\sinh(2RR^{\prime }) \end{array}$$ (18)

$$\begin{array}{ccc} & = & \frac{1}{\sqrt{\pi }}[\exp(-{\left(R-R^{\prime }\right)}^{2})-\exp(-{\left(R+R^{\prime }\right)}^{2})]. \end{array}$$ (19)

For the source concentration,

$$\begin{array}{c} \rho_{\text{source}}(R,t_{n+1})=\chi_{n}F_{n}\text{erfc}(R) \end{array}$$ (20)

where the flux factor *F*_*n*_ is defined as

$$\begin{array}{c} F_{n}=\frac{j_{n}}{8\pi D^{3/2}\Delta t^{1/2}}. \end{array}$$ (21)

Then,

$$\begin{array}{c} \rho (R,t_{n+1})=\rho_{\text{prop}}(R,t_{n+1})+\rho_{\text{source}}(R,t_{n+1}). \end{array}$$ (22)

### Propagation algorithm

The integral in Eq (17) may be evaluated in a number of different ways (e.g., the Fast Gauss Transform [10]). The alternative approach taken here is to find a set of Gauss-diffusion quadrature (GDQ) points ${\left\{x_{\alpha }\right\}}_{\alpha =1}^{N}$ with corresponding weights ${\left\{w_{\alpha }\right\}}_{\alpha =1}^{N}$ that both depend on *R*. Then,

$$\begin{array}{c} \rho (R,t_{n+1})\approx \sum_{\alpha =1}^{N}\rho (x_{\alpha }(R),t_{n})w_{\alpha }(R)+\chi_{n}F_{n}\text{erfc}(R). \end{array}$$ (23)

This, however, leads to an infinite nesting of grid point evaluations:

$$\begin{array}{ccc} \rho (x_{\beta },t_{n+1}) & \approx & \sum_{\alpha =1}^{N}\rho (x_{\alpha }(x_{\beta }),t_{n})w_{\alpha }(x_{\beta })+\chi_{n}F_{n}\text{erfc}(x_{\beta }) \end{array}$$ (24)

$$\begin{array}{ccc} \rho (x_{\alpha }(x_{\beta }),t_{n}) & \approx & \sum_{\gamma =1}^{N}\rho (x_{\gamma }(x_{\alpha }(x_{\beta })),t_{n})w_{\alpha }(x_{\beta })+\chi_{n}F_{n}\text{erfc}(x_{\alpha }(x_{\beta })) \end{array}$$ (25)

$$\begin{array}{cc} & \vdots \end{array}$$ (26)

This is, of course, not desirable because we want a finite, well-defined set of GDQ points {*x*_*α*_}. One can overcome this problem by defining a set of grid points ${\left\{R_{i}\right\}}_{i=1}^{I}$ between which interpolation is used to evaluate the function at the GDQ points. Specifically, for each *R*_*i*_, *ρ*(*R*_*i*_, *t*_*n*+1_) can be calculated from Eq (23) after the {*x*_*α*_(*R*_*i*_)} and {*w*_*α*_(*R*_*i*_)} have been calculated. The interpolation grid and the corresponding GDQ points and weights may be precalculated and saved.

The interpolation used here is a weighted sum of the interpolation points, so that

$$\begin{array}{c} \rho (z)\approx \sum_{j=1}^{I}A_{j}^{\left(z\right)}\rho (R_{j}). \end{array}$$ (27)

The interpolation weights depend on *z* because the *R*_*j*_ are nonuniformly spaced. By Eqs (17) and (23),

$$\begin{array}{ccc} \rho_{\text{prop}}(R_{i},t_{n+1}) & \approx & \sum_{\alpha =1}^{N}\rho (x_{\alpha }(R_{i}),t_{n})w_{\alpha }(R_{i}) \end{array}$$ (28)

$$\begin{array}{ccc} & \approx & \sum_{\alpha =1}^{N}\sum_{j=1}^{I}A_{j}^{\left(x_{\alpha }(R_{i})\right)}\rho (R_{j},t_{n})w_{\alpha }(R_{i}) \end{array}$$ (29)

$$\begin{array}{ccc} & = & \sum_{j=1}^{I}W_{ij}\rho (R_{j},t_{n}) \end{array}$$ (30)

with the combined weights

$$\begin{array}{c} W_{ij}=\sum_{\alpha =1}^{N}A_{j}^{\left(x_{\alpha }(R_{i})\right)}w_{\alpha }(R_{i}). \end{array}$$ (31)

In order to express the iteration process in matrix/vector notation, define the column vector

$$\begin{array}{c} \rho_{n}={\left\{\rho (R_{i},t_{n})\right\}}_{i=1}^{I} \end{array}$$ (32)

and the matrix

$$\begin{array}{c} W={\left\{W_{ij}\right\}}_{i,j=1}^{I}. \end{array}$$ (33)

Then,

$$\begin{array}{c} \rho_{n+1}=W\rho_{n}+\chi_{n}F_{n}s \end{array}$$ (34)

for the source column vector

$$\begin{array}{c} s={\left\{\text{erfc}(R_{i})\right\}}_{i=1}^{I}. \end{array}$$ (35)

One algorithm for evolving *ρ*(*R*, *t*_*n*_) in time then is:

1. Overhead (before the simulation)
  (a) Choose the parameters Δ*t*, the maximum time *T* of the simulation, and the number of GDQ points *N*.
  (b) Calculate the interpolation grid {*R*_*i*_} for this *T*; details are discussed in Subsection Interpolation Grid and Weights.
  (c) Compute the source vector **s** in Eq (35).
  (d) For each *R*_*i*_, compute the GDQ points and weights ${\left\{x_{\alpha }\left(R_{i}\right)\right\}}_{\alpha =1}^{N}$ and ${\left\{w_{\alpha }\left(R_{i}\right)\right\}}_{\alpha =1}^{N}$; details are discussed in Subsection GDQ Points and Appendix C.
  (e) For each *i*, compute the interpolation weights ${\left\{A_{i}^{\left(x_{\alpha }\left(R_{i}\right)\right)}\right\}}_{\alpha =1}^{N}$; details are discussed in Subsection Interpolation Grid and Weights and Appendix A.
  (f) Compute the combined weights matrix *W* using Eq (31).
2. (optional) Perform an error checking simulation; details are discussed in Section Error Analysis.
3. Simulation. Starting with *ρ*(*R*,0) = *c*_0_ *R* for time *t* = 0,
  (a) evaluate the source state *χ*_*n*_ and flux *j*_*n*_, possibly based on the concentrations ***ρ***_*n*_ from the previous time step;
  (b) compute ***ρ***_*n*+1_ from ***ρ***_*n*_ using Eq (34).

The overhead is where all the serious programming work goes, specifically to compute the Gaussian quadrature, the interpolation grid, and the interpolation weights. That having been said, however, none of these steps are as difficult as they first appear to be. First, a *Mathematica* notebook that computes the Gaussian quadrature grid points and weights is available in the Supporting Information (S1 File). Second, programs like *Mathematica* provide function interpolation routines that can be used in the interpolation grid building strategy described in Interpolation Grid and Weights, thereby requiring little actual programming by the user. Lastly, Fornberg [11] provides an easy-to-implement algorithm for interpolation that, with one tweak, is very fast to compute and easily allows the user to change the interpolation order. The overhead calculations are generally fast, taking a few seconds and, if parallelization is used, less than 1 sec.

At each simulation step, the iteration (Eq (34)) requires a matrix/vector multiplication with the *I* × *I* matrix *W*. However, *W* is both small and sparse. Generally, *W* is small because *I* < 200 is generally sufficient for accurate results. This is because the interpolation grid is nonuniformly spaced and chosen based on knowledge of the exact solution of the diffusion problem, thereby *a priori* putting extra points where they are needed (Section Interpolation Grid and Weights). *W* is also sparse because only a small number of neighboring points are used in the Fornberg interpolation. In sample calculations, *W* ranged from 3% to 42% filled, depending on the number of GDQ and Fornberg points, as well as on the spacing of the interpolation grid points (specifically, the number of near and far points, as defined in Interpolation Grid and Weights). The sparsity of *W* and the small size of *W* make the propagation scheme very fast.

### Interpolation grid and weights

There are two components to the interpolation used in the propagation algorithm, namely choosing the points {*R*_*i*_} and choosing an interpolation method. For the latter, we use a method by Fornberg [11] that uses polynomial interpolation between previously chosen points. This was chosen not only because it is easy to implement and quick to compute, but also because changing of the interpolation order *N*_*F*_ (i.e., the order of the interpolating polynomial or, equivalently, the number of nearest-neighbor points around each *R*_*i*_) is effortless for the user. The technical details are given in Appendix A.

The interpolation grid points should be a minimal number of points, and therefore it would be best to have some knowledge of the mathematical structure of the solution *ρ*(*R*, *t*_*n*_). Luckily, it is possible to derive an exact solution (see Appendix B). Specifically,

$$\begin{array}{c} \rho_{n}(R)=\sum_{m=0}^{n}\chi_{n-m}F_{n-m}e_{m}(R) \end{array}$$ (36)

where

$$\begin{array}{c} e_{m}(R)=\{\begin{array}{cc} \text{erfc}(R) & m=0\\ \text{erf}(\frac{R}{\sqrt{m}})-\text{erf}(\frac{R}{\sqrt{m+1}}) & m\geq 1. \end{array} \end{array}$$ (37)

While one can use this exact solution, it is computationally impractical because no work from time *t*_*n*_ can be reused for time *t*_*n*+1_. Therefore, if *T* timesteps have elapsed, it takes *O*(*T* ^2^) operations per location *R* to compute *ρ*(*R*, *T*) via Eq (36), which becomes slower than the propagation method proposed here after ∼ 100 to 1000 timesteps.

Eq (36) does, however, provide useful information for making the interpolation grid. Specifically, one can find an interpolation grid for the functions *e*_*m*_(*R*) for various *m* and taking their union. This will yield points that are located where they are needed most.

The first step is to find the largest possible distance *R*_max_ that could be needed in the simulation that ends at time *T*_max_. This can be determined by considering the worst-case scenario where the current source is on the entire time. *R*_max_ should be chosen so that the concentration there is below some chosen threshold *ɛ*; that is, *R*_max_ is the *r*_max_ that satisfies

$$\begin{array}{c} \frac{j_{\text{max}}}{4\pi D}\frac{1}{r_{\text{max}}}\text{erfc}(\frac{r_{\text{max}}}{2\sqrt{D\Delta t}})=\varepsilon \end{array}$$ (38)

or, in nondimensionalized variables,

$$\begin{array}{c} F_{\text{max}}\text{erfc}(R_{\text{max}})=\varepsilon R_{\text{max}} \end{array}$$ (39)

where *F*_max_ corresponds to the largest possible flux *j*_max_ encountered during the simulation. The interpolation grid then spans [0, *R*_max_].

**Fig 1:**
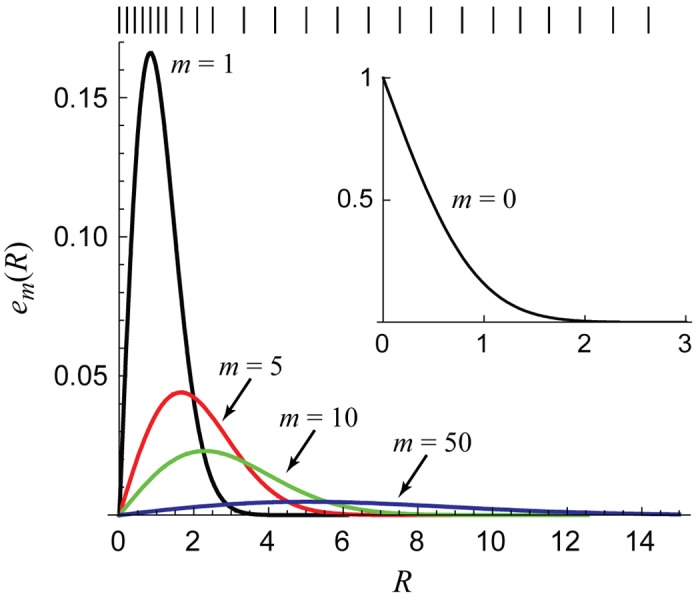
The functions *e*_*m*_(*R*) defined in Eq (37). The hash marks above the main figure are the interpolation grid points for small *I* (Simulation 1 in Table 1).

The second step is to analyze the functions *e*_*m*_(*R*). For each *m* ≥ 1, *e*_*m*_(0) = *e*_*m*_(∞) = 0 and *e*_*m*_(*R*) has one maximum. Moreover, as *m* → ∞, the maximum value decays to 0 and so the *e*_*m*_(*R*) → 0 for all *R*. This is shown in Fig 1. The figure shows that the first few *e*_*m*_(*R*) are the most important numerically and that their contribution is limited to the interval [0, 10] (approximately). We therefore divide the interval [0, *R*_max_] into two parts, the near (*R* ≤ 10) and the far (*R* > 10) intervals. We start with the same uniform grid for each *m*, but the far interval is divided uniformly on a logarithmic scale while the near interval is divided on a linear scale. That is, [0, 10] is divided uniformly while it is [log(10), log(*R*_max_)] that is divided uniformly. This serves two purposes. First, it focuses the points where *e*_1_(*R*) has the most structure (Fig 1) and needs to be resolved the best, namely in the near interval. Second, it keeps the number of grid points to a minimum. Since one can have *R*_max_ > 5000, using the logarithmic scale places relatively few points in the far interval. More points (e.g., by linearly dividing the far interval) are not necessary because the *e*_*m*_(*R*) have little structure in the far interval that needs to be resolved (Fig 1). Also, since *R*_max_ increases with *T*_max_, the number of grid points grows logarithmically with *T*_max_.

After dividing the near and far intervals uniformly, the next step is to interpolate the functions *e*_*m*_(*R*) on these intervals separately. To do that, polynomial interpolation (usually third order) is used on each subinterval of the uniformly divided interval. Specifically, each subinterval is bisected and both *e*_*m*_ and the polynomial approximation are evaluated at this midpoint. Each interval is further bisected until *e*_*m*_ and the interpolation are within a specified tolerance. This is done for many *m* and the union of all these interpolation grids is taken. This does not add many points because each *m* starts with the same uniformly-spaced intervals that are then bisected. Some *m* (especially the small *m*) will have more bisection steps because they have more structure, but this does not affect the accuracy of the *m* that do not have many bisections; in fact it only increases their accuracy. In practice, doing this for *m* up to 500 suffices, with significantly larger *m* adding only a few extra points. Fig 1 shows an example grid. It was generated using the *Mathematica* function FunctionInterpolation, which uses this interpolation scheme.

### GDQ points

Gaussian quadrature integration methods are very efficient when numerically integrating a smooth function *f*(*R*′) against a weight function Ω(*R*′) over integration limits *a* and *b* by using a weighted sum:

$$\begin{array}{c} \int_{a}^{b}f(R^{\prime })\Omega (R^{\prime })dR^{\prime }\approx \sum_{\alpha =1}^{N}f(R_{\alpha })\omega_{\alpha }. \end{array}$$ (40)

The efficiency over standard integration methods (e.g., the trapezoidal rule) comes from being able to choose the locations *R*_*α*_ as well as the weights *ω*_*α*_; standard integration methods prescribe the *R*_*α*_, usually as uniformly-spaced points. With the appropriate choices of *N* Gaussian weights and points, a polynomial of degree 2*N* is integrated exactly [12]. In practice, even a small *N* gives very accurate results if *f* is a very smooth function like we have here (Eq (36) and Fig 1). For the diffusion problem, *N* between 10 and 20 works well (discussed in detail later).

The theory of Gaussian quadratures is well-established (see, for example, Ref. [12]) and for many specific choices of Ω, *a*, and *b* there are standard techniques for computing the Gaussian weights and points for a given *N*. These are generally referred to as, for example, Gauss-Legendre (Ω = 1, *a* = −1, *b* = 1) and Gauss-Laguerre (Ω = *e* ^−*R*′^, *a* = 0, *b* = ∞) quadratures, and so in our case we refer to it as Gauss-diffusion quadrature, or GDQ. Computing a Gaussian quadrature for non-standard weight functions is possible [12], and Appendix C describes in detail how for the diffusion weight function of Eq (18). A *Mathematica* notebook that computes the GDQ weights and points is included in the Supporting Information (S1 File).

## Error analysis

There are several different ways to analyze the accuracy of the propagation technique. By using a concrete example, we next show that it is possible to quantify the error in the simulations using a standardized system. Moreover, that system can also be used to find simulation parameters that achieve a desired accuracy with the shortest computation time.

### Summary of error analysis

Knowing the accuracy of a simulation method is always important, but there are additional reasons for quantifying the error of the propagation method. First, by using interpolation we are introducing errors into the calculation of *ρ*_*n*_ at every time step and using those results to compute *ρ*_*n*+1_. It is therefore a real concern that errors accumulate and lead to first-order errors after the many timesteps required for a simulation. Second, when the flux source is randomly on, *ρ*_*n*_(*R*) can have local maxima and minima that must be resolved accurately. If they are not, then that error may can lead to spurious results at later times. With the example in the next section we show that neither of these occur when the simulation parameters are chosen well. For the vast majority of simulation parameter sets (i.e., *N*, *N*_*F*_, *I*_near_, and *I*_far_) the propagation technique gives very accurate answers, although for some combinations of parameters the *ρ*_*n*_ can diverge. It is therefore important to run these kinds of test simulations.

There are two different checks one can do. The first is a simulation where the flux source is turned on and off randomly and the exact solution in Eq (36) is used to check the result. The second is a simulation where the flux source is always on with a constant flux and the exact solution in Eq (4) is used. Both have pros and cons. With the randomly-on source simulation only relatively small total simulation times *T* can be checked because the time to calculate the exact solution grows as *T* ^2^, as discussed above. However, with this technique one can explicitly see whether the fine details of the *ρ*_*n*_(*R*) profile is reproduced. On the other hand, the advantage of the always-on simulation test is that simulations of arbitrary length can be checked to assure against error accumulation. In the next section, it is shown that the always-on test suffices because it bounds the error; its error is always larger than in the random-on test. One can therefore always run this test to assess simulation accuracy. Moreover, the propagation technique is fast enough that one can test which combination of simulation parameters gives a desired level of accuracy in the fastest time, thereby ensuring accurate results in minimal time. This is important for applications like calcium-induced calcium release were many similar simulations must be done.

### Example

To illustrate this, we consider a specific example and examine the errors produced by different parameter choices. Specifically, the point source had a flux of 10^7^ particles per second, which is of the order of the flux through an ion channel (approximately 1.6 pA of current for a univalent ion). The initial concentration *c*_0_ was 0. The time step was Δ*t* = 10^−7^ seconds and the diffusion coefficient was 10^−9^ m/s^2^, which gives *F*_*n*_ = 6.61 × 10^−5^ M for all time steps. A maximum of 10^6^ time steps were considered for all simulations. Solving Eq (39) with *ɛ* = 10^−17^ gives *R*_max_ = 5148.

All the errors shown here are the maximum absolute difference between the simulated result *ρ*_sim_ and the exact result *ρ*_exact_ over all *R*, scaled by *F*_*n*_:

$$\begin{array}{c} \varepsilon_{\text{sim}}(t)=\frac{1}{F_{n}}\max_{R}|\rho_{\text{sim}}(R,t)-\rho_{\text{exact}}(R,t)|. \end{array}$$ (41)

With this scaling, the exact result is independent of the flux and diffusion coefficient (see Eq (36)). Also, *ρ*_exact_(*R*, *t*)/*F*_*n*_ ≤ 1 for all *R*, and, because it is *O*(1), −log_10_(*ɛ*_sim_) is approximately the number of significant figures the simulation has correct. It is important to note, however, that this may not be the error metric of choice for a given problem. In this example, for instance, having a relatively large error with *ɛ*_sim_ = 10^−3^ corresponds to a *concentration* error of 66.1 nM (*F*_*n*_ *ɛ*_sim_), and if nothing in the problem is sensitive to nanomolar concentrations, then having a stricter error requirement like *ɛ*_sim_ = 10^−5^ is overkill and probably a waste of computational time and effort.

**Fig 2:**
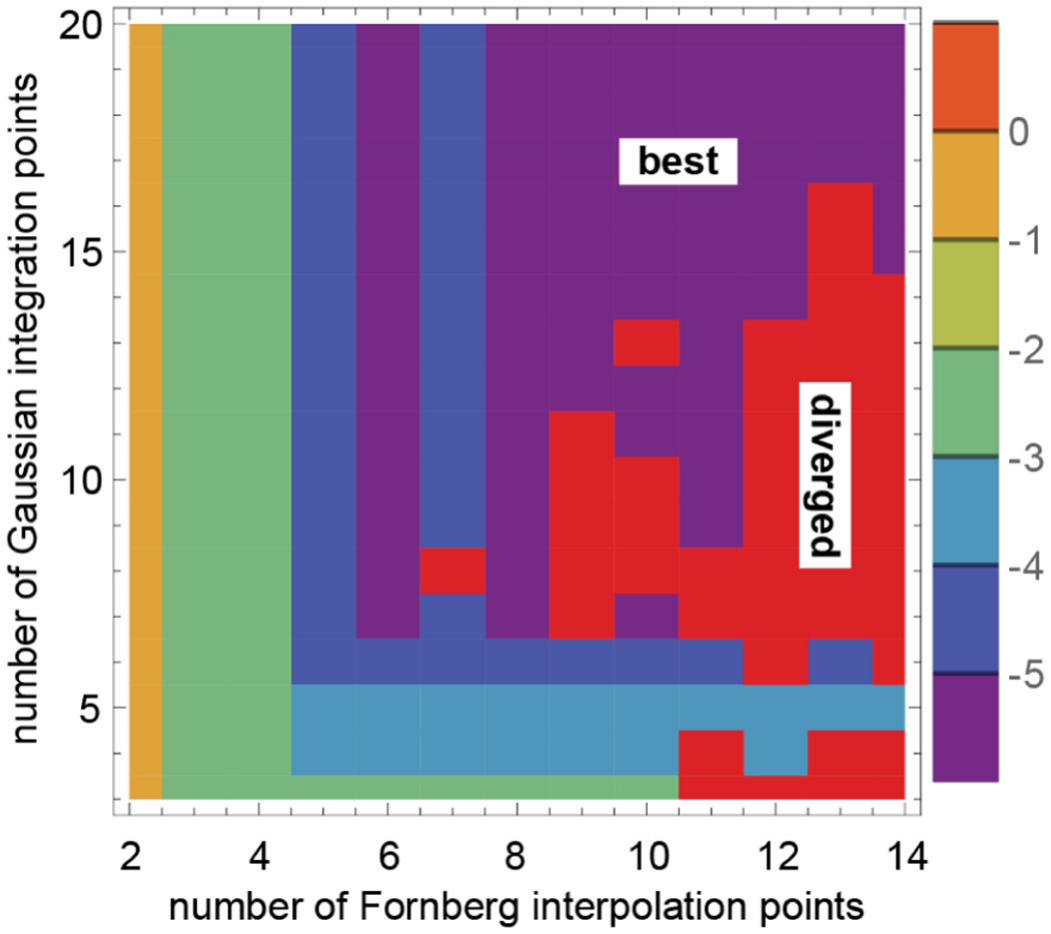
Error for different *N*_*F*_ (*x*-axis) and *N* (*y*-axis) for simulations lasting 10^4^ time steps. The flux source was always on. The maximum difference between the simulated *ρ* and exact solution at all *R* over all 10^4^ time steps (max_*t*_{*ɛ*_sim_(*t*)}) is shown on a color-coded log_10_ scale. Errors larger than 1 were truncated to 1 to make the graph more readable.

As a first test, we consider different *N* (the number of Gaussian integration points) and *N*_*F*_ (the number of Fornberg interpolation points) using 173 interpolation grid points (80 near points for *R* ≤ 10 and 73 far points for *R* > 10). The results for simulations lasting 10^4^ time steps are shown in Fig 2. The flux source was always on. In general, as *N* increases, the error decreases. However, the same is not always true for *N*_*F*_. Usually the error decreases for *N*_*F*_ ≤ 8, but for larger *N*_*F*_ the simulation *ρ* can diverge (e.g., values > 10^10^). This is not unexpected, however, as high interpolation order does not necessarily produce high interpolation accuracy. For example, a constant function is well-approximated by linear interpolation (*N*_*F*_ = 1), but poorly by high-order polynomials because these necessarily require oscillations between the grid points in order to be 0 at the grid points. This is, in fact, what occurs here; the interpolation oscillates far from the flux source where *ρ* is constant (at 0). As the simulation continues, these oscillations are either damped out for the best results (purple in Fig 2) or amplified for the diverged results (red) (data not shown). If divergence does occur, the user should be aware that small changes in *N* and *N*_*F*_ can alleviate this problem (Fig 2).

**Fig 3:**
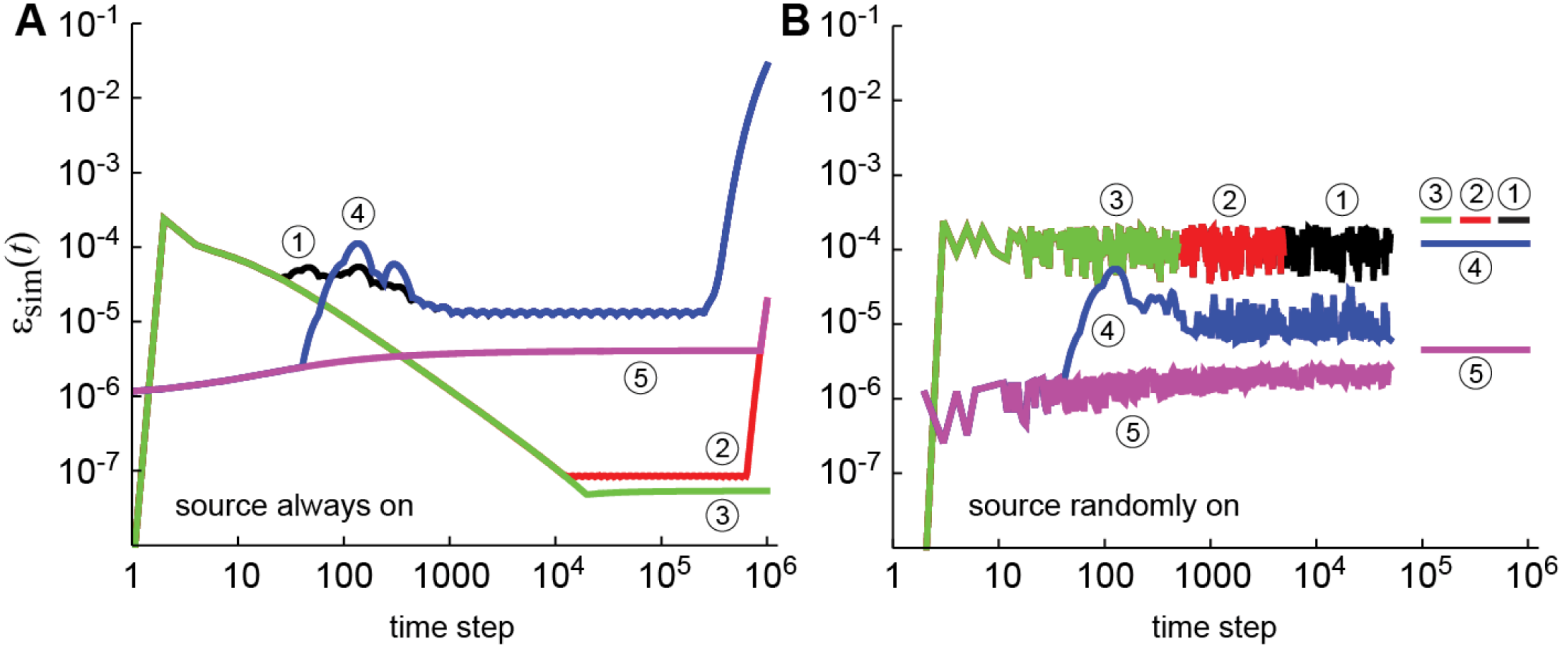
Error versus simulation time for five of the six simulations listed in Table 1. The circled numbers correspond to the Simulation listed in the table. (A) The flux source is always on. (B) The flux source is randomly on for 50000 time steps. The bars on the right side are the maximum error over 50000 time steps from panel A to show that the error in the always-on simulations bounds the error of the randomly-on simulations. For all simulations, *N* = 20 and *N*_*F*_ = 9.

With *N* = 20 and *N*_*F*_ = 9 that produce accurate results, as a second test we consider the number of interpolation grid points *I*. More specifically, we consider how the number of near points *I*_near_ (for 0 ≤ *R* ≤ 10, as discussed in Interpolation Grid and Weights) and the number of far points *I*_far_ (*R* > 10) affect the error. Table 1 lists the six cases that were considered, and Fig 3 shows *ɛ*_sim_(*t*) for the first five cases; the results of Simulation 6 were identical to Simulation 5 except that in Fig 3A it did not have the upswing in error near the 10^6^-th time step that occurs in Simulation 5 (and overlaps with Simulation 2).

**Table 1:** Parameters used in Fig 3. The circled numbers in Fig 3 correspond to the simulation number (Sim.) in the Table. For each simulation, the number of near (*I*_near_) and far (*I*_far_) interpolation grid points are shown, as well as the sum (*I*). Simulation 6 is not shown in Fig 3 because it was the same as Simulation 5 except that it did not have the uptick in error at the end of the simulation. For all simulations, *N* = 20 and *N*_*F*_ = 9.

Sim.	*I*_near_	*I*_far_	*I*
1	19	45	64
2	19	90	109
3	19	180	199
4	83	51	134
5	83	90	173
6	83	180	263

We first analyze the case where the flux source is on at all times (Fig 3A). To make sense of the results, consider Simulations 1, 2, and 3, which differ in the number of far points. At the beginning of the simulation, they overlap. At later times, they separate and eventually Simulations 1 and 2 begin to diverge. This indicates that increasing number of far points increases accuracy at later times. This is consistent with the results of Simulations 4, 5, and 6. Again, they overlap at early times and separate according to how many far points there are; the more far points, the smaller the late-time error. Conversely, the more near points, the smaller the early-time error. For example, compare Simulations 1 and 4 and Simulations 2 and 5. Intuitively, these results make sense: early in the simulation *ρ* is changing near the source so the near interval is most important, while late in the simulation *ρ* is changing in the far interval.

**Fig 4:**
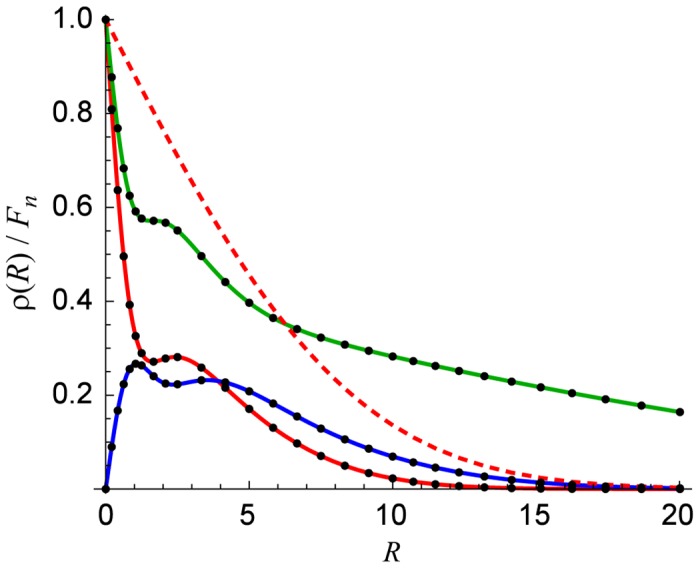
*ρ*(*R*) for a randomly-on flux source at various simulation times. It illustrates both the complicated structure of these curves and to show how the interpolation grid points are more dense in the regions that need them. The points are the simulation results and the curves are the exact result. For comparison, the dashed curve is the always-on exact result at the same time step as the red curve. For clarity a very small number of interpolation grid points were used (*I* = 64).

In the case just considered, the flux source is always on. However, the propagation technique is designed for fluctuating sources. Moreover, the always-on case does not require the specially-constructed interpolation grid. This is shown in Fig 4 where *ρ* profiles at different times are shown for a randomly-on flux source. For comparison, the dashed line shows the always-on result at the same time step as the red solid curve. The random-on profiles can have multiple local maxima and inflection points, something not seen for the monotonically decaying always-on profile. Because of the more complicated *ρ* profiles, it is possible that the error for the randomly-on case is much different than for the always-on case. For example, not resolving those maxima or minima at one time can propagate an error to later times.

Fig 3B shows that this is not the case. In fact, for all six cases considered, the maximum error is always less for the random-on case than for the always-on case. This is very useful because the exact answer is much easier to compute for the always-on case; for the random-on case only about 5 × 10^5^ time steps can be computed in a reasonable amount of time. Bounding the error is also important because it makes it always possible to pre-compute the error; if the details of how the flux source turns on or off are unimportant for measuring simulation error, then one can use the always-on case.

**Fig 5:**
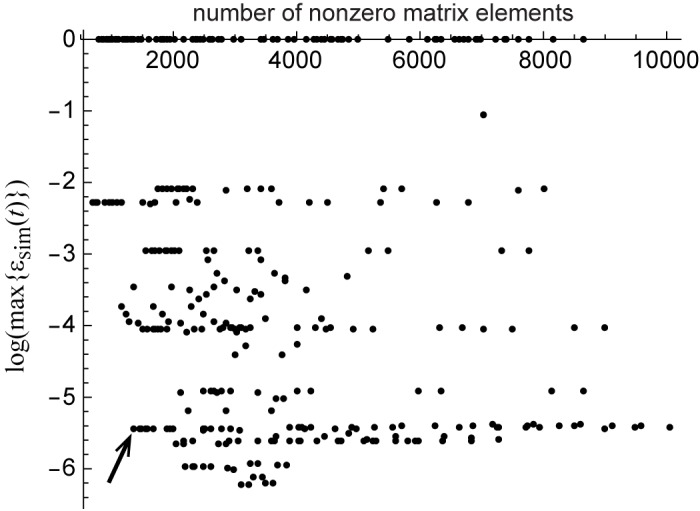
Simulation error versus the number of nonzero elements in *W* (to represent computation time). Each point is for a different parameter set (*N*, *N*_*F*_, *I*_near_, and *I*_far_) for a long simulation of 10^6^ time steps for the example described in the main text with the flux source always on. The arrow points to the optimal balance of high accuracy and computation speed. Errors larger than 1 were truncated to 1 to make the graph more readable.

Moreover, one can optimize accuracy versus computation speed by combining all of these error checks. The computation speed of the propagation method is dominated by the matrix/vector multiplication in Eq (34), and because *W* is sparse, the number of multiplications required is the number of nonzero entries in *W*. Therefore, one can vary all the parameters above (*N*, *N*_*F*_, *I*_near_, and *I*_far_) and plot max_*t*_{*ɛ*_sim_(*t*)} versus the number of nonzero entries in *W*. The fastest and most accurate set of parameters can then be chosen for all future simulations. Fig 5 shows this for 336 parameter choices for 10^6^ time steps. The arrow points to the parameter set with the fastest computation time that still achieve high accuracy (max_*t*_{*ɛ*_sim_(*t*)} ≤ 10^−5^), in this case *N* = 10, *N*_*F*_ = 8, *I*_near_ = 25, and *I*_far_ = 90 with *W* having 1356 nonzero entries. Because the propagation method is fast, it is possible to scan this large set of parameters; Fig 5 took approximately 7 hours on a desktop computer with a 6-core 3.33 GHz i7-980X processor using *Mathematica* version 9 (Wolfram Research, Champaign, Illinois), which could have been significantly faster if done in parallel rather than in series. This time investment is well worth it for applications where a large number of simulations must be done because it guarantees both high accuracy and the fastest overall computation speed for production runs.

To see how this compares to classical methods like BTCS and Crank-Nicolson, one must first consider their mathematical structure. Each discretizes both the time and space derivatives in Eq (1), almost always with uniform spacing Δ*t* and Δ*r* for the time and space coordinates, respectively. This creates a system of linear equations to be solved at each time step with a tridiagonal matrix [12]. Since tridiagonal systems can be solved with *O*(*N*_*r*_) operations (*N*_*r*_ is the number of spatial grid points), to be comparable to the propagation method, *N*_*r*_ should be around 1000.

*N*_*r*_ is determined by both Δ*r* and the maximum distance *L* to be considered. Since the diffusion equation requires both initial and boundary conditions at *r* = 0 and *r* = *L*, one must pick *L* large enough so that the concentration is (approximately) 0. For the example in this section, *R*_max_ = 5148 corresponds to *L* = 102.96 *μ*m. From Fig 4 one can see that it takes a resolution in *R* of at least 0.25, which corresponds to 5 nm for the example in this section. Therefore, *N*_*r*_ ≡ ⌈*L*/Δ*r*⌉ = 20592. Even for such a large system, a simple Crank-Nicolson implementation produced max_*t*_{*ɛ*_sim_(*t*)} > −4.1, significantly worse than many propagation implementations with suboptimal parameters (Fig 5) and 6 to 45 times as many nonzero matrix entries.

## Extension to reaction-diffusion

The focus so far has been on the diffusion equation with a point source (Eq (1)). However, the work done so far can also be extended to include a simple model where chemical reactions remove the diffusing particles. If we assume that these buffer molecules are present at high concentrations and do not change in time or space, then Eq (1) becomes [13]

$$\begin{array}{c} \frac{\partial c(r,t)}{\partial t}=\frac{D}{r^{2}}\frac{\partial }{\partial r}(r^{2}\frac{\partial c}{\partial r}(r,t))+s(r,t)+k_{-}B-k_{+}bc(r,t) \end{array}$$ (42)

where *k*_−_ is the off-rate of the buffer and *k*_+_ is the on-rate. Since we assume the buffer is present at high concentration, the free buffer concentration *b* and the bound buffer concentration *B* are known constants (see, for example, [13]). Eq (6) then has two new source and sink terms whose form is the same as Eq (8). For the source term from the diffusant unbinding from the buffer,

$$\begin{array}{ccc} c_{-}(r,t_{n+1}) & = & k_{-}B\int_{t_{n}}^{t_{n+1}}\int_{{\mathbb{R}}^{3}}{\left(4\pi D(t_{n+1}-t^{\prime })\right)}^{-3/2}\exp(-\frac{{\left|r-r^{\prime }\right|}^{2}}{4D(t_{n+1}-t^{\prime })})dr^{\prime }dt^{\prime } \end{array}$$ (43)

$$\begin{array}{ccc} & = & k_{-}B\int_{t_{n}}^{t_{n+1}}\frac{dt^{\prime }}{\sqrt{D(t_{n+1}-t^{\prime })}} \end{array}$$ (44)

$$\begin{array}{ccc} & = & 2k_{-}B\sqrt{\frac{\Delta t}{D}} \end{array}$$ (45)

where Eq (14) with a constant concentration was used to obtain the intermediate equation. For the sink term from the diffusant binding to buffer,

$$\begin{array}{ccc} c_{+}(r,t_{n+1}) & = & k_{+}b\int_{t_{n}}^{t_{n+1}}\int_{{\mathbb{R}}^{3}}{\left(4\pi D(t_{n+1}-t^{\prime })\right)}^{-3/2}\exp(-\frac{{\left|r-r^{\prime }\right|}^{2}}{4D(t_{n+1}-t^{\prime })})c(r^{\prime },t^{\prime })dr^{\prime }dt^{\prime } \end{array}$$ (46)

$$\begin{array}{ccc} & \approx & k_{+}b\Delta t\int_{{\mathbb{R}}^{3}}{\left(4\pi D\Delta t\right)}^{-3/2}\exp(-\frac{{\left|r-r^{\prime }\right|}^{2}}{4D\Delta t})c(r^{\prime },t_{n})dr^{\prime } \end{array}$$ (47)

$$\begin{array}{ccc} & = & k_{+}b\Delta t\cdot c_{\text{prop}}(r,t_{n+1}) \end{array}$$ (48)

where the intermediate equation used a one-point quadrature for the time integral and the final result follows from Eq (7).

All of the results of the previous sections then carry forward directly:

$$\begin{array}{c} \rho (R,t_{n+1})=(1-k_{+}b\Delta t)\rho_{\text{prop}}(R,t_{n+1})+\rho_{\text{source}}(R,t_{n+1})+4k_{-}B\Delta t\cdot R \end{array}$$ (49)

and Eq (34) becomes

$$\begin{array}{c} \rho_{n+1}=(1-k_{+}b\Delta t)W\rho_{n}+s_{n}^{\prime } \end{array}$$ (50)

where

$$\begin{array}{c} s_{n}^{\prime }=\chi_{n}F_{n}s+4k_{-}B\Delta tR \end{array}$$ (51)

with

$$\begin{array}{c} R={\left\{R_{i}\right\}}_{i=1}^{I}. \end{array}$$ (52)

## Conclusion

An integration quadrature for the propagation integral of the spherically-symmetric diffusion equation from a time-dependent point source was developed. This integral (Eq (17)) was evaluated using a new Gauss-diffusion quadrature and a specialized interpolation grid was used to find the values at the Gaussian quadrature points for the next integration. This scheme then balances accuracy with speed by using a small number of integration and interpolation grid points to achieve high accuracy over many time steps.

The analysis of this propagation technique shows that it has a number of positive attributes:

1. It works with a small number of grid points. Even with just 115 interpolation grid points one can get very accurate results even for long simulations (e.g., 10^6^ time steps in Fig 5) with a propagation matrix with < 1400 nonzero entries.
2. The update step is a simple sparse-matrix/vector multiplication.
3. The simulation error is quantifiable before any “real” simulations are done.
4. Simulation error and computation time can be optimized together to minimize both error and simulation time.
5. The time step Δ*t* can be arbitrarily large, being defined only by the rate at which the flux source turns on and off.
6. It is unconditionally stable.
7. It can easily incorporate nonlinear feedback into the flux source, for example, from the concentration profile calculated at previous time steps.

The algorithm developed here is an alternative to more traditional finite difference or finite element approaches. It is different since it computes the concentration profile at the next time step using a simple sparse-matrix/vector multiplication rather than a (sparse) matrix solve needed for unconditionally-stable implicit-time methods. Differences in computation speed then depend on the number of grid points used in the traditional approaches and the specifics of the matrix solution method, making it difficult to compare the methods head-to-head. However, if uniform spatial discretization is used in classical methods like BTCS or Crank-Nicolson, they produce matrices with significantly more nonzero entries than the propagation method. The matrix/vector multiplication of the propagation method is similar to that of conditionally-stable explicit-time methods, except that these methods require small time steps to be convergent, while the time step for the propagation method is solely determined by the timescale on which the flux source turns on and off. However, the propagation method is meant to be an alternative technique that tries to minimize the number of points (and nonzero matrix entries) while retaining high accuracy.

## Appendix A: Revised Fornberg algorithm

So that the paper is self-contained, a brief summary of Fornberg’s interpolation algorithm [11] is given, noting one time-saving change and focusing only on function interpolation, rather than derivatives as well; that is, here *M* = 0 in Fornberg’s notation. If there are grid points ${\left\{R_{i}\right\}}_{i=0}^{I-1}$ to interpolate from, then for some given point *x*_0_ we seek an approximation of *f*(*x*_0_) as a weighted average of *N*_*F*_ ≤ *I* of these interpolation points:

$$\begin{array}{c} f(x_{0})\approx \sum_{\nu =0}^{N_{F}-1}A_{\nu }^{\left(x_{0}\right)}f(R_{\nu }^{*}) \end{array}$$ (53)

where the ${\left\{R_{\nu }^{*}\right\}}_{\nu =0}^{N_{F}-1}$ is the subset of *N*_*F*_ interpolation points closest to *x*_0_. The superscript (*x*_0_) indicates that the weights *A*_*ν*_ are different for different *x*_0_.

It is important to note that the Fornberg algorithm uses only the first *N*_*F*_ number of interpolation points given to it. Therefore, for each *x*_0_ one must input the *N*_*F*_ interpolation points nearest to *x*_0_. In particular, for each *x*_0_ a different set of interpolation points must be given; if one always inputs {*R*_*i*_}, then the first *N*_*F*_ of them will always be used to compute *f*(*x*_0_), even if *x*_0_ is not close to any of these points. Hence the $R_{\nu }^{*}$ notation in Eq (53). This is a point not made clearly by Fornberg.

The Fornberg algorithm for function interpolation only (i.e., not evaluating derivatives) is:

set all *a*_*k*, *l*_ = 0 for 0 ≤ *k*, *l* ≤ *N*_*F*_ − 1 and *a*_0,0_ = 1

set *c*_1_ = 1

for *n* = 1 to *N*_*F*_ − 1 (this is the time-saving change; Fornberg has *I* instead of *N*_*F*_ − 1 which results in an extremely long calculation of many 0’s)

  set *c*_2_ = 1

  for *ν* = 1 to *N*_*F*_ − 2

   set *c*_3_ = *R*_*n*_ − *R*_*ν*_

   set *c*_2_ = *c*_2_ *c*_3_

   set *a*_*n*,*ν*_ = (*R*_*n*_ − *x*_0_)*a*_*n*−1,*ν*_/*c*_3_

  next *ν*

  set $a_{n,n}=-\frac{c_{1}}{c_{2}}\left(R_{n-1}-x_{0}\right)a_{n-1,n-1}$

  set *c*_1_ = *c*_2_

next *n*

set $A_{\nu }^{\left(x_{0}\right)}=a_{N_{F}-1,\nu }$

## Appendix B: Derivation of the exact solution

Here, a brief derivation of the exact solution to the propagation integral evolution is given, specifically that

$$\begin{array}{ccc} \rho_{n}(R) & = & \sum_{m=1}^{n}\chi_{n-m}F_{n-m}[\text{erf}(\frac{R}{\sqrt{m}})-\text{erf}(\frac{R}{\sqrt{m+1}})]\\ & & +\chi_{n}F_{n}E(R) \end{array}$$ (54)

where

$$\begin{array}{c} E(R)=\text{erfc}(R). \end{array}$$ (55)

While this result is almost surely not new, it was derived independently by the author in the following way.

Define

$$\begin{array}{c} {\bar{\rho }}_{n}(R)=\{\begin{array}{cc} \rho_{n}(R) & \text{if}\;R\geq 0\\ -\rho_{n}(-R) & \text{if}\;R<0 \end{array} \end{array}$$ (56)

and note that the weight function of Eq (19) has the property

$$\begin{array}{c} W(R,-R^{\prime })=W(-R,R^{\prime })=-W(R,R^{\prime }). \end{array}$$ (57)

Then

$$\begin{array}{c} {\bar{\rho }}_{n}(R)=\frac{1}{2}\int_{-\infty }^{\infty }{\bar{\rho }}_{n-1}(R^{\prime })W(R,R^{\prime })dR^{\prime }+\chi_{n}F_{n}E(R). \end{array}$$ (58)

Taking the Fourier transform (denoted with ˜ and 𝓕_*R*_ to be explicit of the variable *R*), we get

$$\begin{array}{c} {\tilde{\bar{\rho }}}_{n}(\omega )=\frac{1}{2}\int_{-\infty }^{\infty }{\bar{\rho }}_{n-1}(R^{\prime }){𝓕}_{R}(W(R,R^{\prime }))dR^{\prime }+\chi_{n}F_{n}\tilde{E}(\omega ) \end{array}$$ (59)

with

$$\begin{array}{c} {𝓕}_{R}(W(R,R^{\prime }))=\frac{1}{\sqrt{2\pi }}e^{-\omega^{2}/4}(e^{i\omega R^{\prime }}-e^{-i\omega R^{\prime }}). \end{array}$$ (60)

Therefore, with * denoting the complex conjugate,

$$\begin{array}{ccc} {\tilde{\bar{\rho }}}_{n}(\omega ) & = & \frac{1}{2}e^{-\omega^{2}/4}({\tilde{\bar{\rho }}}_{n-1}(\omega )-{\tilde{\bar{\rho }}}_{n-1}{\left(\omega \right)}^{*})+\chi_{n}F_{n}\tilde{E}(\omega ) \end{array}$$ (61)

$$\begin{array}{ccc} & = & ie^{-\omega^{2}/4}\text{Im}({\tilde{\bar{\rho }}}_{n-1}(\omega ))+\chi_{n}F_{n}\tilde{E}(\omega ). \end{array}$$ (62)

Assuming the initial concentration is 0, we have

$$\begin{array}{c} \rho_{0}(R)=\chi_{0}F_{0}E(R) \end{array}$$ (63)

and

$$\begin{array}{c} {\tilde{\bar{\rho }}}_{0}(\omega )=\chi_{0}F_{0}f_{0}(\omega ) \end{array}$$ (64)

where

$$\begin{array}{c} f_{0}(\omega )=\sqrt{\frac{2}{\pi }}\frac{1-e^{-\omega^{2}/4}}{\omega }. \end{array}$$ (65)

Using Eq (62) repeatedly, we find that

$$\begin{array}{c} \text{Im}({\tilde{\bar{\rho }}}_{n}(\omega ))=\sum_{m=0}^{n}\chi_{n-m}F_{n-m}e^{-m\omega^{2}/4}f_{0}(\omega ). \end{array}$$ (66)

Using the fact that

$$\begin{array}{c} {𝓕}^{-1}(ie^{-m\omega^{2}/4}f_{0}(\omega ))=\text{erf}(\frac{R}{\sqrt{m}})-\text{erf}(\frac{R}{\sqrt{m+1}}), \end{array}$$ (67)

taking the inverse Fourier transform of Eq (62) gives that

$$\begin{array}{ccc} \rho_{n}(R) & = & {𝓕}^{-1}(ie^{-\omega^{2}/4}\text{Im}({\tilde{\bar{\rho }}}_{n-1}(\omega )))+\chi_{n}F_{n}E(R) \end{array}$$ (68)

$$\begin{array}{ccc} & = & \sum_{m=0}^{n-1}\chi_{n-1-m}F_{n-1-m}{𝓕}^{-1}(ie^{-(m+1)\omega^{2}/4}f_{0}(\omega ))+\chi_{n}F_{n}E(R) \end{array}$$ (69)

$$\begin{array}{ccc} & = & \sum_{m=1}^{n}\chi_{n-m}F_{n-m}[\text{erf}(\frac{R}{\sqrt{m}})-\text{erf}(\frac{R}{\sqrt{m+1}})]+\chi_{n}F_{n}E(R) \end{array}$$ (70)

which is the same as Eq (36).

## Appendix C: Gaussian quadrature

The theory of Gaussian quadratures is developed in a number of texts (e.g., that by Press et al. [12]) so only a brief outline relevant to the diffusion problem is given. One must compute the coefficients *a*_*j*_ (*j* = 0, 1, …, *N*) and *b*_*j*_ (*j* = 1, …, *N*) for the orthonormal polynomials

$$\begin{array}{ccc} p_{-1}(x) & = & 0 \end{array}$$ (71)

$$\begin{array}{ccc} p_{0}(x) & = & 1 \end{array}$$ (72)

$$\begin{array}{ccc} p_{j+1}(x) & = & \left(x-a_{j})p_{j}(x)-b_{j}p_{j-1}(x\right) \end{array}$$ (73)

where

$$\begin{array}{c} a_{j}(R)=\frac{\left\langle xp_{j}{\left(x\right)}^{2}\right\rangle }{\left\langle p_{j}{\left(x\right)}^{2}\right\rangle } \end{array}$$ (74)

and

$$\begin{array}{c} b_{j}(R)=\frac{\left\langle p_{j}{\left(x\right)}^{2}\right\rangle }{\left\langle p_{j-1}{\left(x\right)}^{2}\right\rangle } \end{array}$$ (75)

for the inner product

$$\begin{array}{c} \langle f\rangle =\int_{0}^{\infty }f(x)W(R,x)dx. \end{array}$$ (76)

Note that because the integral’s weight function depends on *R*, the coefficients depend on *R* as well.

To determine the coefficients *a*_*j*_ and *b*_*j*_, we need the moments of the weight function:

$$\begin{array}{ccc} M_{n} & \equiv & \int_{0}^{\infty }{\left(R^{\prime }\right)}^{n}\cdot W(R,R^{\prime })dR^{\prime } \end{array}$$ (77)

$$\begin{array}{ccc} & = & \frac{2}{\sqrt{\pi }}R\exp(-R^{2})\Gamma (\frac{n}{2}+1)\Phi (\frac{n}{2}+1,\frac{3}{2};R^{2}) \end{array}$$ (78)

where Φ(*α*, *γ*;*z*) is the confluent hypergeometric function which is equal to the generalized hypergeometric series _1_ *F*_1_(*α*;*γ*;*z*) [14]. This function has the recurrence relationship [14]

$$\begin{array}{c} \Phi (\alpha +1,\gamma ;z)=\frac{z+2\alpha -\gamma }{\alpha }\Phi (\alpha ,\gamma ;z)+\frac{\gamma -\alpha }{\alpha }\Phi (\alpha -1,\gamma ;z) \end{array}$$ (79)

that will allow us to evaluate the moments efficiently. From the structure of this recurrence relationship, it is easiest to break the moments into even *n* and odd *n*.

For even *n* = 2*k* (*k* ≥ 1) with *α* = *n*/2 = *k*, we have

$$\begin{array}{c} \Phi (k+1,\frac{3}{2};R^{2})=\frac{R^{2}+2k-\frac{3}{2}}{k}\Phi (k,\frac{3}{2};R^{2})+\frac{\frac{3}{2}-k}{k}\Phi (k-1,\frac{3}{2};R^{2}) \end{array}$$ (80)

or, when

$$\begin{array}{c} \phi_{k}=\Phi (k,\frac{3}{2};R^{2}), \end{array}$$ (81)

$$\begin{array}{c} \phi_{k+1}=\frac{R^{2}+2k-\frac{3}{2}}{k}\phi_{k}+\frac{\frac{3}{2}-k}{k}\phi_{k-1}. \end{array}$$ (82)

This gives a procedure for increasing *k* by starting with

$$\begin{array}{c} \phi_{0}=1 \end{array}$$ (83)

and

$$\begin{array}{c} \phi_{1}=\frac{\sqrt{\pi }}{2}\frac{\exp(R^{2})}{R}\text{erf}(R). \end{array}$$ (84)

Using that

$$\begin{array}{c} \Gamma (\frac{n}{2}+1)=\Gamma (k+1)=k!, \end{array}$$ (85)

we get

$$\begin{array}{ccc} M_{0} & = & \frac{2}{\sqrt{\pi }}R\exp(-R^{2})\cdot \phi_{1} \end{array}$$ (86)

$$\begin{array}{ccc} & = & \text{erf}(R) \end{array}$$ (87)

and

$$\begin{array}{ccc} M_{2} & = & \frac{2}{\sqrt{\pi }}R\exp(-R^{2})\cdot \phi_{2} \end{array}$$ (88)

$$\begin{array}{ccc} & = & \frac{2}{\sqrt{\pi }}R\exp(-R^{2})\cdot ((R^{2}+\frac{1}{2})\phi_{1}+\frac{1}{2}) \end{array}$$ (89)

$$\begin{array}{ccc} & = & \left(R^{2}+\frac{1}{2})M_{0}+\frac{1}{\sqrt{\pi }}R\exp(-R^{2}\right) \end{array}$$ (90)

$$\begin{array}{ccc} & = & (R^{2}+\frac{1}{2})\text{erf}(R)+\frac{1}{\sqrt{\pi }}R\exp(-R^{2}), \end{array}$$ (91)

so that for even *n* ≥ 4

$$\begin{array}{ccc} M_{2k} & = & \frac{2}{\sqrt{\pi }}R\exp(-R^{2})\cdot k!\phi_{k+1} \end{array}$$ (92)

$$\begin{array}{ccc} & = & \frac{2}{\sqrt{\pi }}R\exp(-R^{2})\cdot k!(\frac{R^{2}+2k-\frac{3}{2}}{k}\phi_{k}+\frac{\frac{3}{2}-k}{k}\phi_{k-1}) \end{array}$$ (93)

$$\begin{array}{ccc} & = & \frac{2}{\sqrt{\pi }}R\exp(-R^{2})\cdot (k-1)!((R^{2}+2k-\frac{3}{2})\phi_{k}+(\frac{3}{2}-k)\phi_{k-1}) \end{array}$$ (94)

$$\begin{array}{ccc} & = & (R^{2}+2k-\frac{3}{2})\cdot M_{2(k-1)}+(k-1)(\frac{3}{2}-k)\cdot M_{2(k-2)}. \end{array}$$ (95)

Here Eq (95) is derived by substituting in Eq (92).

For odd *n* = 2*l* + 1 (*l* ≥ 1) with *α* = *l* + 1/2, we have

$$\begin{array}{c} \Phi (l+\frac{3}{2},\frac{3}{2};R^{2})=\frac{R^{2}+2l-\frac{1}{2}}{l+\frac{1}{2}}\Phi (l+\frac{1}{2},\frac{3}{2};R^{2})+\frac{1-l}{l+\frac{1}{2}}\Phi (l-\frac{1}{2},\frac{3}{2};R^{2}) \end{array}$$ (96)

so that

$$\begin{array}{c} \phi_{l+3/2}=\frac{R^{2}+2l-\frac{1}{2}}{l+\frac{1}{2}}\phi_{l+1/2}+\frac{1-l}{l+\frac{1}{2}}\phi_{l-1/2}. \end{array}$$ (97)

This gives a procedure for increasing *l* by using

$$\begin{array}{c} \Gamma (\frac{n}{2}+1)=\Gamma (l+1+\frac{1}{2})=\frac{\sqrt{\pi }}{2^{l+1}}(2l+1)!! \end{array}$$ (98)

and starting with

$$\begin{array}{ccc} M_{1} & = & \frac{2}{\sqrt{\pi }}R\exp(-R^{2})\cdot \frac{\sqrt{\pi }}{2}\phi_{3/2} \end{array}$$ (99)

$$\begin{array}{ccc} & = & R \end{array}$$ (100)

and

$$\begin{array}{ccc} M_{3} & = & \frac{2}{\sqrt{\pi }}R\exp(-R^{2})\cdot \frac{3\sqrt{\pi }}{4}\Phi (\frac{5}{2},\frac{3}{2};R^{2}) \end{array}$$ (101)

$$\begin{array}{ccc} & = & \frac{3}{2}R(\frac{2}{3}R^{2}+1) \end{array}$$ (102)

$$\begin{array}{ccc} & = & \frac{1}{2}R(2R^{2}+3) \end{array}$$ (103)

so that

$$\begin{array}{ccc} M_{2l+1} & = & \frac{2}{\sqrt{\pi }}R\exp(-R^{2})\cdot \frac{\sqrt{\pi }}{2^{l+1}}(2l+1)!!\cdot \phi_{l+3/2} \end{array}$$ (104)

$$\begin{array}{ccc} & = & R\exp(-R^{2})\cdot \frac{1}{2^{l}}(2l+1)!!(\frac{R^{2}+2l-\frac{1}{2}}{l+\frac{1}{2}}\phi_{l+1/2}+\frac{1-l}{l+\frac{1}{2}}\phi_{l-1/2}) \end{array}$$ (105)

$$\begin{array}{ccc} & = & \frac{2^{l-1}}{2^{l}}\frac{(2l+1)!!}{(2l-1)!!}\frac{R^{2}+2l-\frac{1}{2}}{l+\frac{1}{2}}M_{2l-1}+\frac{2^{l-2}}{2^{l}}\frac{(2l+1)!!}{(2l-3)!!}\frac{1-l}{l+\frac{1}{2}}M_{2l-3} \end{array}$$ (106)

$$\begin{array}{ccc} & = & \frac{1}{2}(2l+1)\frac{R^{2}+2l-\frac{1}{2}}{l+\frac{1}{2}}M_{2l-1}+\frac{1}{4}(4l^{2}-1)\frac{1-l}{l+\frac{1}{2}}M_{2l-3} \end{array}$$ (107)

$$\begin{array}{ccc} & = & (R^{2}+2l-\frac{1}{2})M_{2(l-1)+1}-(l-\frac{1}{2})(l-1)M_{2(l-2)+1}. \end{array}$$ (108)

Here, Eq (107) is derived by substituting in Eq (104).

These moments can then be used to build a Gaussian quadrature using standard procedures, namely finding the eigenvalues {*λ*_*j*_} and eigenvectors {**v**_*j*_} of the symmetric tridiagonal matrix [12]

$$\begin{array}{c} (\begin{array}{ccccc} a_{0} & \sqrt{b_{1}} & 0 & \cdots & 0\\ \sqrt{b_{1}} & a_{1} & \sqrt{b_{2}} & \ddots & 0\\ 0 & \ddots & \ddots & \ddots & 0\\ 0 & \ddots & \sqrt{b_{N-2}} & a_{N-2} & \sqrt{b_{N-1}}\\ 0 & \cdots & 0 & \sqrt{b_{N-1}} & a_{N-1} \end{array}). \end{array}$$ (109)

Then, the Gaussian grid points are the eigenvalues (i.e., *x*_*α*_ = *λ*_*α*_) and the weights are

$$\begin{array}{ccc} w_{\alpha } & = & v_{\alpha ,0}^{2}\cdot \int_{0}^{\infty }W(R,R^{\prime })dR^{\prime } \end{array}$$ (110)

$$\begin{array}{ccc} & = & v_{\alpha ,0}^{2}\cdot \text{erf}(R) \end{array}$$ (111)

where *v*_*α*,0_ is the first element of **v**_*α*_.

From a theoretical point of view this is straightforward. From a computational point of view, however, it is important to note that the inner product ratios that define the coefficients *a*_*j*_ and *b*_*j*_ in Eqs (74) and (75) must be done with extremely high precision (e.g., 100 digits or more of working precision) or round-off errors accumulate quickly, even for relatively small *N*. Moreover, the matrix in Eq (109) is extreme ill-conditioned. With similarly high working precision, however, finding the eigenvalues and eigenvectors is fast and effective. Lastly it is noted that computing the inner products,

$$\begin{array}{c} \langle x^{a}p_{j}{\left(x\right)}^{2}\rangle \equiv \int_{0}^{\infty }W(R,x)x^{a}{\left(\sum_{n}c_{n}^{\left(j\right)}x^{n}\right)}^{2}dx \end{array}$$ (112)

(*a* = 0 or 1) can be done by working only with the coefficients $c_{n}^{\left(j\right)}$ of the polynomial *p*_*j*_ to find the coefficients of *p*_*j*_(*x*)^2^ by using a list convolution and then using a dot product with the vector of moments *M*_*n*_.

This procedure gives GDQ points ${\left\{x_{\alpha }\left(R\right)\right\}}_{\alpha =1}^{N}$ and weights ${\left\{w_{\alpha }\left(R\right)\right\}}_{\alpha =1}^{N}$. Note that while the matrix and inner product operations must be done with many digits of accuracy, once the GDQ points and weights are calculated they can be truncated to machine precision for the diffusion calculations. A *Mathematica* notebook that computes the GDQ points and weights is available in the Supporting Information (S1 File).

An alternative approach to calculating the GDQ points and weights is the procedure described by Golub and Welsch [15], but ill-conditioning will still be an issue.

## Supporting Information

S1 File Mathematica notebook containing code to compute the GDQ points and weights as described in Appendix C. (NB)
